# Supplementary material for: Peptide transporter structure reveals binding and action mechanism of a potent PEPT1 and PEPT2 inhibitor
Source: Commun Chem. 2022 Feb 24;5:23. doi: 10.1038/s42004-022-00636-0 (PMC9814568; doi:10.1038/s42004-022-00636-0)
Supplement: Supplementary file 2 — Supplementary Information [file 42004_2022_636_MOESM2_ESM.pdf]

Supplementary Information for:

Peptide transporter structure reveals binding and action mechanism of a potent PEPT1 and PEPT2 inhibitor

Mirko Stauffer[1,*], Jean-Marc Jeckelmann[1,*], Hüseyin Ilgü[1], Zöhre Ucurum[1], Rajendra Boggavarapu[1,2] and Dimitrios Fotiadis[1,+]

[1] Institute of Biochemistry and Molecular Medicine, and Swiss National Centre of Competence in Research (NCCR) TransCure, University of Bern, Bern, Switzerland

[2] Present address: Department of Physiology and Biophysics, Case Western Reserve University, Cleveland, OH, USA

[*] These authors contributed equally

[+] Corresponding author

Correspondence and requests for materials should be addressed to D.F. (email: dimitrios.fotiadis@ibmm.unibe.ch)

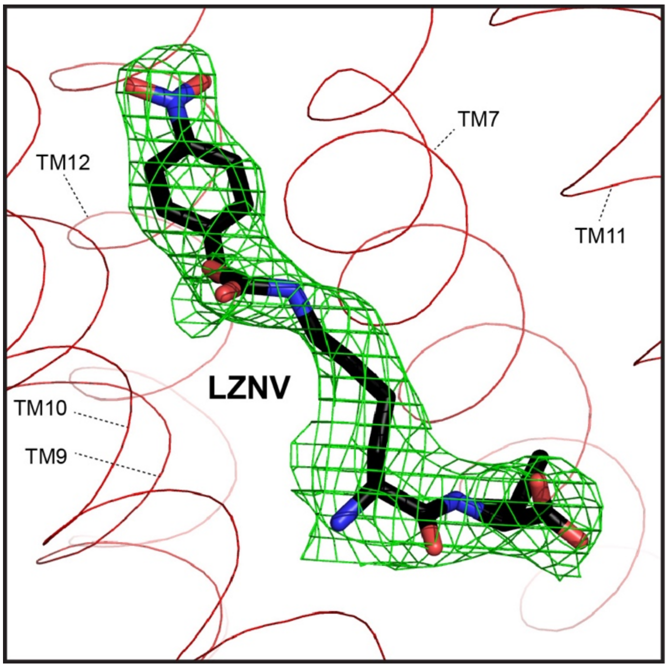

Supplementary Figure 1 Polder OMIT map and LZNV bound YePEPT^{K314A} structure. The polder OMIT map is illustrated as green mesh and contoured at 3.0 σ. The YePEPT^{K314A} protein is displayed as red ribbon and the LZNV molecule as black sticks and transmembrane helices (TMs) shown are labelled.

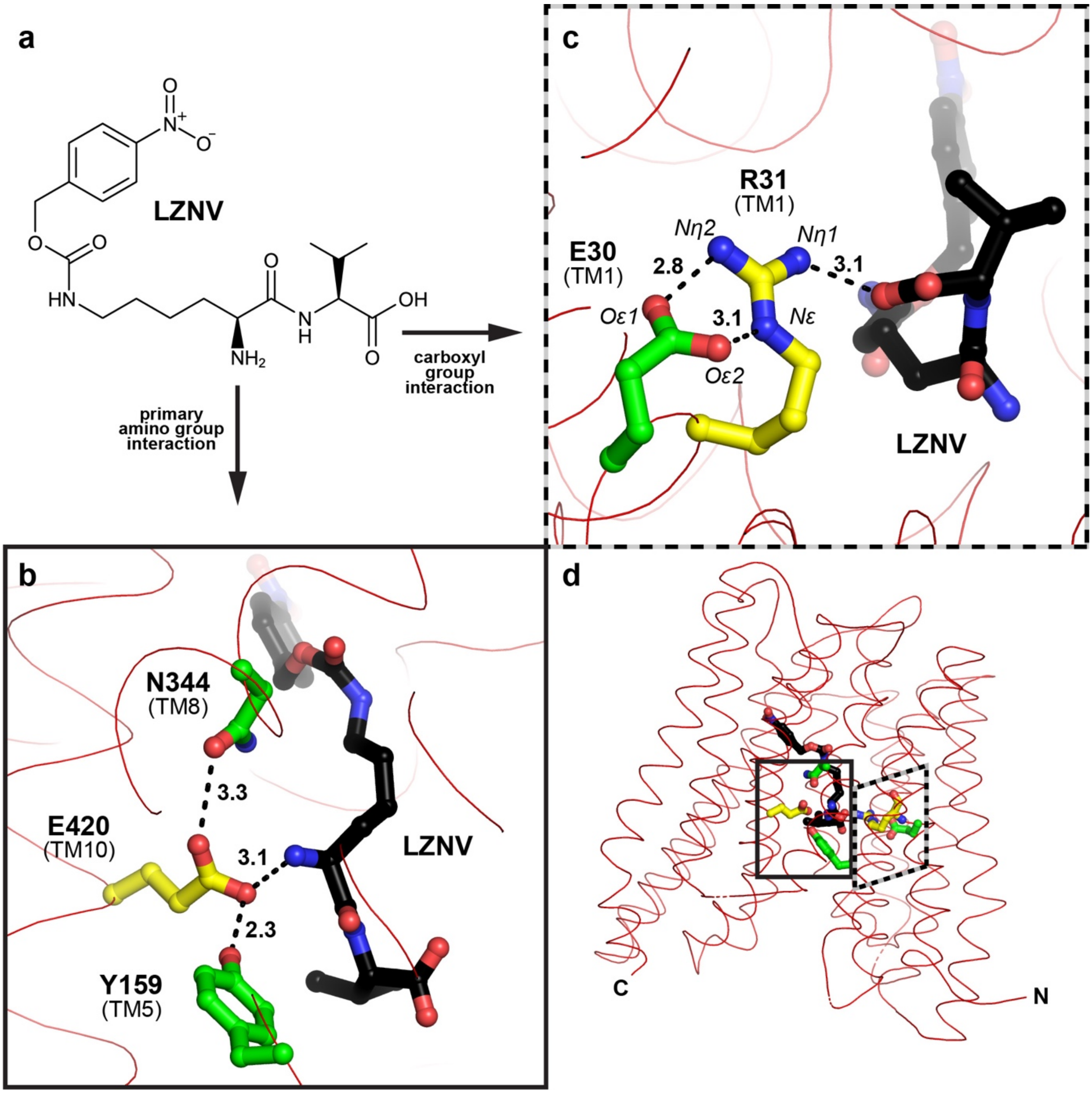

Supplementary Figure 2 Spatial orientation of amino acid side chains involved in LZNV binding. **a** Chemical structure of LZNV. Arrows indicate the interactions of the amino group with E420 (**b**) and the carboxyl group with R31 (**c**). **b** Interactions of E420 with Y159 and N344. **c** Interactions of R31 with E30. In **b** and **c**, interactions are shown as dashed lines (distances in Å), amino acids are labelled in the one-letter code and the corresponding transmembrane helices (TMs) are indicated in brackets. **d** Location of the Interactions of E420 (black box) and R31 (dashed parallelogram) in the structure of LZNV bound YePEPT^{K314A}. LZNV is shown as black sticks, LZNV binding side chains and orientating side chains as yellow and green sticks, respectively.

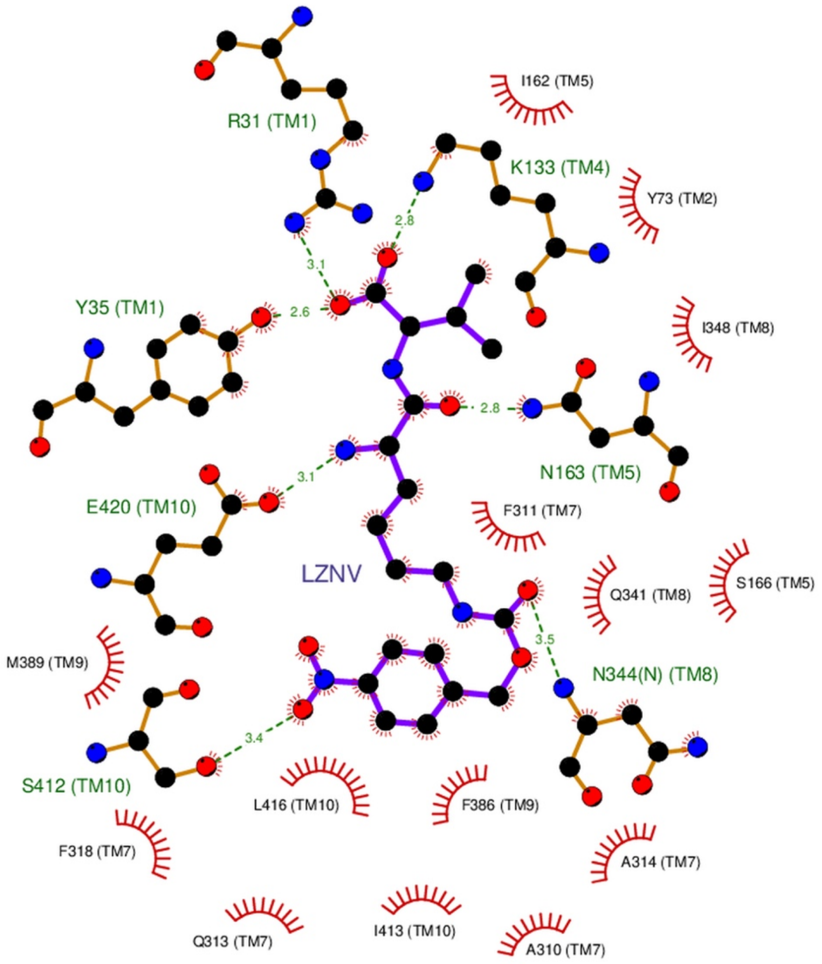

Supplementary Figure 3 Schematic 2D representation of protein-LZNV interactions. Polar and ionic interactions within a distance ≤ 3.5 Å (green dashed lines, distances in Å) and hydrophobic interactions within a distance ≤ 3.9 Å (red sunbursts) from amino acid residues to LZNV (purple) are displayed. Amino acids are labelled in the one-letter code and corresponding transmembrane helices (TMs) are indicated in brackets. For N344, the interaction with the main-chain nitrogen is indicated by N344(N). The figure was prepared using LigPlot+[1].

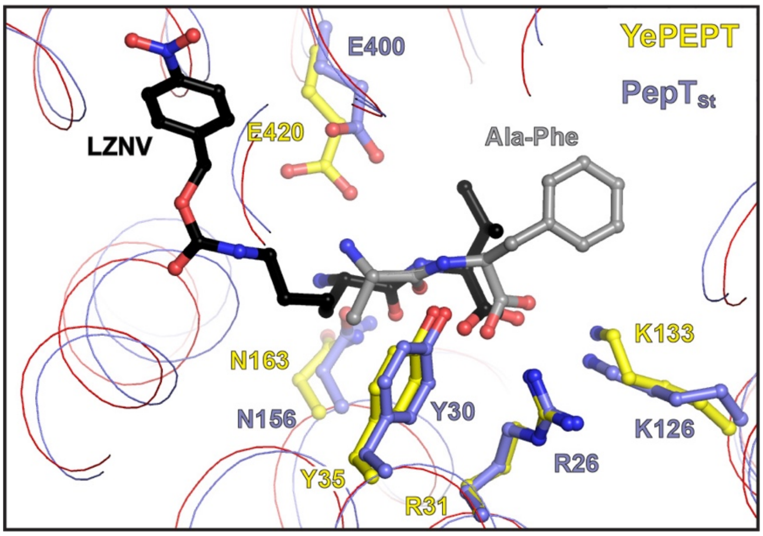

Supplementary Figure 4 Conserved dipeptide backbone binding mode. The structures of LZNV bound YePEPT^{K314A} and Ala-Phe bound PepT$_{St}$ (peptide transporter from *Streptococcus thermophilus*; PDB ID 4D2C[2]) were aligned (r.m.s.d = 1.144 Å for 302 Cα atoms) and are displayed. A view into the ligand binding site indicates that the conserved dipeptide backbone binding mode is retained in LZNV binding to YePEPT^{K314A}. Conserved amino acid side chains involved in dipeptide backbone binding are shown as sticks and are labelled. YePEPT^{K314A} and PepT$_{St}$ side chains are coloured in yellow and bright blue. The ligands LZNV and Ala-Phe are coloured in black and grey.

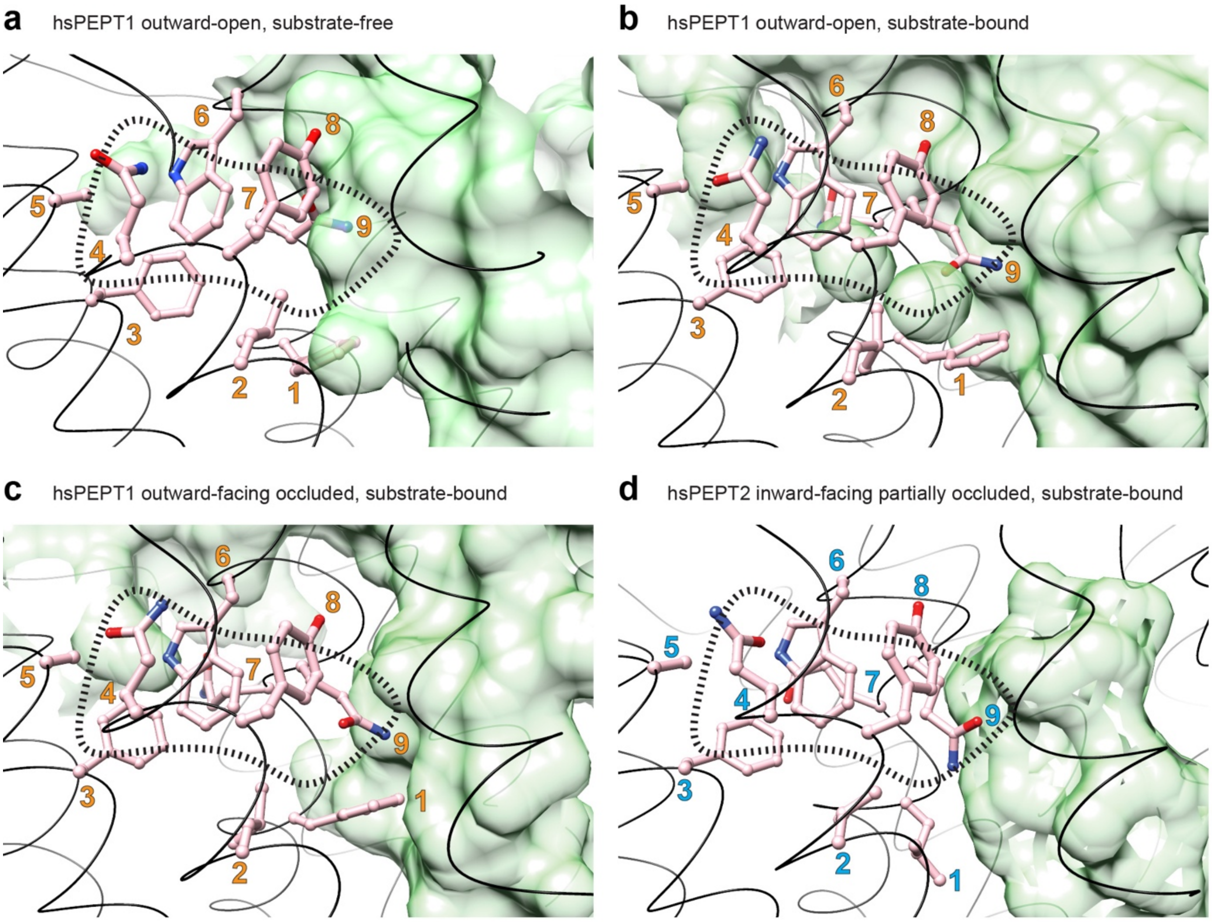

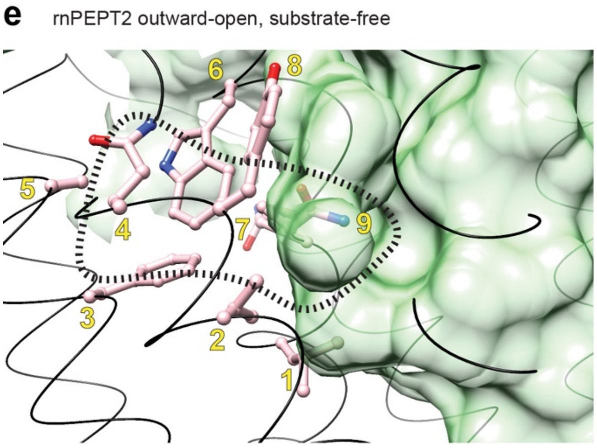

h

Residue	hsPEPT1	hsPEPT2 & rnPEPT2
1	F297	L316
2	L591	V618
3	F372	F391
4	Q587	Q614
5	A375	A394
6	W304	W323
7	Q299	Q318
8	Y588	Y615
9	Q300	Q319

Supplementary Figure 5 Solvent accessibility in the PZ pocket locations of mammalian PEPT1 and PEPT2. Displayed are ribbons (black lines), amino acid side chains (pink sticks) and solvent filled cavities (light green surfaces) of the currently available experimental human (hs) and rat (rn) PEPT1 and PEPT2 structures in different conformational and, substrate-free or -bound states: **a** hsPEPT1 outward-open, substrate-free (PDB ID 7PN1[3]), **b** hsPEPT1 outward-open, substrate-bound (PDB ID 7PMX[3]), **c** hsPEPT1 outward-facing occluded, substrate-bound (PDB ID 7PMW[3]), **d** hsPEPT2 inward-facing partially occluded, substrate-bound (PDB ID 7PMY[3]) and **e** rnPEPT2 outward-open, substrate-

free (PDB ID 7NQK[4]). The PZ pocket residues displayed are according to the alignment given in Supplementary Table 2. In panels a-e these residues are labelled 1-9 and corresponding amino acid one-letter codes listed in panel **h**. The PZ pocket locations are marked by dashed connected lines. Solvent filled cavities were calculated employing the CASTp server[5]. Structures were visualized with UCSF Chimera[6].

Supplementary Table 1. Data collection, processing and refinement statistics

**Data collection and processing**	LZNV bound[a]	Apo
Beamline	X06SA, Swiss Light Source - SLS	X06SA, Swiss Light Source - SLS
Detector	Eiger 16M	Pilatus 6M
Wavelentgh (Å)	1.0	1.0
Space group	$P2_12_12_1$	$P2_12_12_1$
Unit-cell: *a*, *b*, *c* (Å); $\alpha = \beta = \gamma$ (°)	88.4, 100.4, 100.1; 90	89.9, 101.1, 103.7; 90
Anisotropy direction[b]		
overall (Å)	**2.66**	**2.93**
along h axis (Å)	3.18	3.15
along k axis (Å)	2.63	**2.71**
along l axis (Å)	**2.54**	2.86
Resolution (Å)[c]	45.85-2.54 (2.66-2.54)	46.12-2.71 (2.84-2.71)
Measured reflections	269,227 (13,296)	120,611 (6,030)
Unique reflections	24,643 (1,158)	21,272 (1,065)
Redundancy	10.9 (11.5)	5.7 (5.7)
R_{meas}[d]	0.12 (2.36)	0.09 (1.67)
$R_{p.i.m.}$[e]	0.05 (0.93)	0.05 (0.93)
$CC_{1/2}$[f]	0.99 (0.54)	1.00 (0.42)
Mean $I/\sigma(I)$	19.7 (1.2)	14.9 (1.2)
Completeness (%)[g]	95.1 (79.3)	92.8 (75.1)
**Refinement**		
Resolution (Å)	24.96-2.54	28.81-2.71
R_{work} / R_{free}[h] (%)	26.71 / 29.14	26.04 / 29.22
No. of atoms	3,827	3,681
Protein / Ligand	3,749 / 78	3,681 / 0
Mean *B* factor (Å^2)	95.3	80.6
Protein / Ligand	95.0 / 115.6	80.6 / 0
RMSD		
Bond length (Å)	0.008	0.007
Bond angle (°)	1.129	1.065
Ramachandran plot (%)		
Favoured region	98.1	98.1
Allowed region	100	100
Disallowed region	0	0

Values in parentheses are for the highest resolution shell.

[a] Datasets from 2 crystals were merged.

[b] The anisotropic resolution limits were computed with AIMLESS[7] based on $CC_{1/2} > 0.50$.

[c] The statistics reflect data truncated by STARANISO (http://staraniso.globalphasing.org/) to remove poorly measured reflections affected by anisotropy.

[d] R_{meas} as defined by Diederichs and Karplus (1997)[8].

[e] Precision-indicating merging *R* factor $R_{p.i.m}$ as defined by Weiss (2001)[9].

[f] $CC_{1/2}$ is the Pearson correlation coefficient of two-half data sets as described by Karplus and Diederichs (2012)[10].

[g] The completeness after the anisotropic correction was obtained by least-square fitting an ellipsoid to the reciprocal lattice points at the cut-off surface defined by a local mean $I/\sigma I$ threshold of 1.2, rejecting outliers in the fit due to spurious deviations, and calculating the fraction of observed data lying inside the ellipsoid.

[h] Random 5% reflections from working set were excluded from refinement for R_{free} calculation.

Supplementary Table 2 Amino acid residues forming the PZ pocket in different bacterial and mammalian POTs

YePEPT^{K314A}	PepT$_{St}$	GkPOT	PepT$_{So}$	PepT$_{So2}$	PepT$_{Xc}$	PepT$_{Sh}$	DtpA	DtpD	hsPEPT1	rnPEPT1	hsPEPT2	rnPEPT2
F311	E299	Q309	F315	Y291	F410	Q310	Y292	A284	F297	F297	L316	L316
Q313	Q301	Q311	Q317	Q293	Q422	Q312	Q294	Q286	Q299	Q299	Q318	Q318
A314	G302	G312	K318	M294	K423	G313	M295	G287	Q300	Q300	Q319	Q319
F318	L306	L316	W322	L298	W427	L317	L299	I291	W304	W304	W323	W323
F386	F368	F382	W387	F371	W492	Y387	F364	F359	F372	F372	F391	F391
M389	M371	I385	V390	Y374	V495	I390	P368	T363	A375	A375	A394	A394
S412	S392	S405	P411	G394	P516	S410	S388	G383	Q587	Q589	Q614	Q614
I413	W393	Y406	Y412	Y395	Y517	Y411	Y389	L384	Y588	Y590	Y615	Y615
L416	V396	V409	L415	Y398	L520	C414	Q392	M387	L591	L593	V618	V618

The multiple sequence alignment was performed using Clustal Omega[11]. Origins (organisms), abbreviations and Uniprot accession codes of listed POTs: *Streptococcus thermophilus* (PepT$_{St}$, Uniprot assecion code: Q5M4H8), *Geobacillus kaustophilus* (GkPOT, Q5KYD1), *Shewanella oneidensis* (PepT$_{So}$, Q8EKT7; PepT$_{So2}$, Q8EHE6), *Xanthomonas campestris* (PepT$_{Xc}$, Q8PAS2), *Staphylococcus hominis* (PepT$_{Sh}$, A0A657M1C3), *Escherichia coli* (DtpA, P77304; DtpD, P75742), *Homo sapiens* (hsPEPT1, P46059; hsPEPT2, Q16348) and *Rattus norvegicus* (rnPEPT1, P51574; rnPEPT2, Q63424).

Supplementary References

1. Laskowski, R. A. & Swindells, M. B. LigPlot+: Multiple ligand–protein interaction diagrams for drug discovery. *J. Chem. Inf. Model.* **51**, 2778–2786 (2011).
2. Lyons, J. A. *et al.* Structural basis for polyspecificity in the POT family of proton-coupled oligopeptide transporters. *EMBO Rep.* **15**, 886–893 (2014).
3. Killer, M., Wald, J., Pieprzyk, J., Marlovits, T. C. & Löw, C. Structural snapshots of human PepT1 and PepT2 reveal mechanistic insights into substrate and drug transport across epithelial membranes. *Sci. Adv.* (2021).
4. Parker, J. L. *et al.* Cryo-EM structure of PepT2 reveals structural basis for proton-coupled peptide and prodrug transport in mammals. *Sci. Adv.* **7**, eabh3355 (2021).
5. Tian, W., Chen, C., Lei, X., Zhao, J. & Liang, J. CASTp 3.0: computed atlas of surface topography of proteins. *Nucleic Acids Res.* **46**, W363–W367 (2018).
6. Pettersen, E. F. *et al.* UCSF Chimera - A visualization system for exploratory research and analysis. *J. Comput. Chem.* **25**, 1605–1612 (2004).
7. Evans, P. R. & Murshudov, G. N. How good are my data and what is the resolution? *Acta Crystallogr. Sect. D Biol. Crystallogr.* **69**, 1204–1214 (2013).
8. Diederichs, K. & Karplus, P. A. Improved R-factors for diffraction data analysis in macromolecular crystallography. *Nat. Struct. Biol.* **4**, 269–275 (1997).
9. Weiss, M. S. Global indicators of X-ray data quality. *J. Appl. Crystallogr.* **34**, 130–135 (2001).
10. Karplus, P. A. & Diederichs, K. Linking crystallographic model and data quality. *Science* **336**, 1030–1033 (2012).
11. Sievers, F. *et al.* Fast, scalable generation of high-quality protein multiple sequence alignments using Clustal Omega. *Mol. Syst. Biol.* **7**, 539 (2011).
