# Supplementary material for: Peptide transporter structure reveals binding and action mechanism of a potent PEPT1 and PEPT2 inhibitor
Source: Commun Chem. 2022 Feb 24;5:23. doi: 10.1038/s42004-022-00636-0 (PMC9814568; doi:10.1038/s42004-022-00636-0)
Supplement: Supplementary file 3 — Reporting Summary [file 42004_2022_636_MOESM3_ESM.pdf]

nature research

Corresponding author(s): Prof. Dr. Dimitrios Fotiadis

Last updated by author(s): Jan 18, 2022

Reporting Summary

Nature Research wishes to improve the reproducibility of the work that we publish. This form provides structure for consistency and transparency in reporting. For further information on Nature Research policies, see our Editorial Policies and the Editorial Policy Checklist.

Statistics

For all statistical analyses, confirm that the following items are present in the figure legend, table legend, main text, or Methods section.

n/a	Confirmed	
☐	☒	The exact sample size (n) for each experimental group/condition, given as a discrete number and unit of measurement
☐	☒	A statement on whether measurements were taken from distinct samples or whether the same sample was measured repeatedly
☒	☐	The statistical test(s) used AND whether they are one- or two-sided<br>*Only common tests should be described solely by name; describe more complex techniques in the Methods section.*
☒	☐	A description of all covariates tested
☒	☐	A description of any assumptions or corrections, such as tests of normality and adjustment for multiple comparisons
☒	☐	A full description of the statistical parameters including central tendency (e.g. means) or other basic estimates (e.g. regression coefficient) AND variation (e.g. standard deviation) or associated estimates of uncertainty (e.g. confidence intervals)
☒	☐	For null hypothesis testing, the test statistic (e.g. F, t, r) with confidence intervals, effect sizes, degrees of freedom and P value noted<br>*Give P values as exact values whenever suitable.*
☒	☐	For Bayesian analysis, information on the choice of priors and Markov chain Monte Carlo settings
☒	☐	For hierarchical and complex designs, identification of the appropriate level for tests and full reporting of outcomes
☒	☐	Estimates of effect sizes (e.g. Cohen's d, Pearson's r), indicating how they were calculated

Our web collection on statistics for biologists contains articles on many of the points above.

Software and code

Policy information about availability of computer code

Data collection

Datasets of YePEPT-K314A were collected at the X06SA (PXI) beamline of the Swiss Light Source (SLS; Paul Scherrer Institute, Villigen, Switzerland) using either a PILATUS 6M (apo YePEPT-K314A) or an EIGER 16M (LZNV bound YePEPT-K314A) detector (Dectris).

Data analysis

Structure determination:
For the LZNV bound YePEPT-K314A structure, two datasets originating from different crystals were indexed and integrated with XDS and the data merged using the BLEND program, which is part of the CCP4 program suite. For data analysis of the apo YePEPT-K314A structure, a dataset originating from one crystal was indexed and integrated with XDS. From that point on data analysis of the two independent datasets was carried out in the same manner, i. e., datasets were processed by the STARANISO software (http://staraniso.globalphasing.org/) to account for the anisotropic nature of the diffraction data. The structures were solved by molecular replacement using the coordinates of YePEPT-wildtype (PDB ID code 4W6V) applying PHASER. After iterative cycles of manual model building using COOT and structure refinement runs applying phenix.refine the final structures of LZNV-bound and apo YePEPT-K314A were obtained. Figures involving structures were prepared using PyMol (The PyMol Molecular Graphics System; Schrödinger).

Uptake and TSA experiments:
Data were analysed and plotted using Prism Graphpad 6.

For manuscripts utilizing custom algorithms or software that are central to the research but not yet described in published literature, software must be made available to editors and reviewers. We strongly encourage code deposition in a community repository (e.g. GitHub). See the Nature Research guidelines for submitting code & software for further information.

Data

Policy information about availability of data

All manuscripts must include a data availability statement. This statement should provide the following information, where applicable:

- Accession codes, unique identifiers, or web links for publicly available datasets
- A list of figures that have associated raw data
- A description of any restrictions on data availability

Relevant data are available from the corresponding author on reasonable request. Atomic coordinates for the apo- and LZNV bound crystal structures of YePEPT-K314A have been deposited in the Protein Data Bank under accession numbers 7Q0L (apo) and 7Q0M (LZNV bound).

Field-specific reporting

Please select the one below that is the best fit for your research. If you are not sure, read the appropriate sections before making your selection.

☒ Life sciences ☐ Behavioural & social sciences ☐ Ecological, evolutionary & environmental sciences

For a reference copy of the document with all sections, see nature.com/documents/nr-reporting-summary-flat.pdf

Life sciences study design

All studies must disclose on these points even when the disclosure is negative.

Sample size	TSA experiments: Presented data originate from at least three independent experiments.<br>Uptake experiments: Presented data originate from three independent experiments, each at least in triplicates.
Data exclusions	TSA and uptake experiments: No data was excluded.
Replication	TSA and uptake experiments were each repeated at least three times.<br>For X-ray crystallization several hundred crystals were measured.
Randomization	Randomization is not necessary and was thus not applied.
Blinding	Blinding was not applied as it is not necessary in the field of X-ray crystallography.

Reporting for specific materials, systems and methods

We require information from authors about some types of materials, experimental systems and methods used in many studies. Here, indicate whether each material, system or method listed is relevant to your study. If you are not sure if a list item applies to your research, read the appropriate section before selecting a response.

Materials & experimental systems

n/a	Involved in the study	
☒	☐	Antibodies
☒	☐	Eukaryotic cell lines
☒	☐	Palaeontology and archaeology
☒	☐	Animals and other organisms
☒	☐	Human research participants
☒	☐	Clinical data
☒	☐	Dual use research of concern

Methods

n/a	Involved in the study	
☒	☐	ChIP-seq
☒	☐	Flow cytometry
☒	☐	MRI-based neuroimaging
